# SARS-CoV-2 RNA in exhaled air of hospitalized COVID-19 patients

**DOI:** 10.1038/s41598-022-13008-4

**Published:** 2022-05-30

**Authors:** Lisa Kurver, Corné H. van den Kieboom, Kjerstin Lanke, Dimitri A. Diavatopoulos, Gijs J. Overheul, Mihai G. Netea, Jaap ten Oever, Reinout van Crevel, Karin Mulders-Manders, Frank L. van de Veerdonk, Heiman Wertheim, Jeroen Schouten, Janette Rahamat-Langendoen, Ronald P. van Rij, Teun Bousema, Arjan van Laarhoven, Marien I. de Jonge

**Affiliations:** 1 Department of Internal Medicine and Radboud Centre for Infectious Diseases, Radboud University Medical Center, 6525 GA Nijmegen, The Netherlands; 2 Laboratory of Medical Immunology, Radboud Institute for Molecular Life Sciences, Radboud University Medical Center, Geert Grooteplein Zuid 10, route 469, Nijmegen, 6525 GA The Netherlands; 3 Department of Medical Microbiology, Radboud Institute for Molecular Life Sciences, Radboud University Medical Center, 6525 GA Nijmegen, The Netherlands; 4 Department of Immunology and Metabolism, Life and Medical Sciences Institute, University of Bonn, 53115 Bonn, Germany; 5 Department of Intensive Care Medicine, Radboud University Medical Center, 6525 GA Nijmegen, The Netherlands; 6 Department of Medical Microbiology, Radboud Centre for Infectious Diseases, Radboud University Medical Center, 6525 GA Nijmegen, The Netherlands; 7 Xheal Diagnostics B.V, 6525 GC Nijmegen, The Netherlands

**Keywords:** SARS-CoV-2, Viral infection

## Abstract

Knowledge about contagiousness is key to accurate management of hospitalized COVID-19 patients. Epidemiological studies suggest that in addition to transmission through droplets, aerogenic SARS-CoV-2 transmission contributes to the spread of infection. However, the presence of virus in exhaled air has not yet been sufficiently demonstrated. In pandemic situations low tech disposable and user-friendly bedside devices are required, while commercially available samplers are unsuitable for application in patients with respiratory distress. We included 49 hospitalized COVID-19 patients and used a disposable modular breath sampler to measure SARS-CoV-2 RNA load in exhaled air samples and compared these to SARS-CoV-2 RNA load of combined nasopharyngeal throat swabs and saliva. Exhaled air sampling using the modular breath sampler has proven feasible in a clinical COVID-19 setting and demonstrated viral detection in 25% of the patients.

## Introduction

SARS-CoV-2 transmission is thought to largely depend on droplets arising from the upper respiratory tract, expelled through talking, coughing, and sneezing, which settle down quickly and relatively close to its source^1^. However, aerosols, in which the virus remains replicative for at least three hours, remain suspended in the air drifting long distances^2–5^, suggesting a role for transmission via aerosols. This was first confirmed by Richard et al*.*, showing that SARS-CoV-2 can be transmitted via air between ferrets^6^. The role of superspreading events further suggests that aerosol transmission contributes to the pandemic^7^. It was recently found that the newly emerged variants of concern show increased infectivity and further confirming the importance of aerosol-mediated spread^8–10^.

Nasopharyngeal throat swabs are a common diagnostic sample, and it is challenging to effectively sample exhaled virions^11^. Given the multiple modes of transmission, it remains questionable whether the SARS-CoV-2 RNA load in the upper respiratory tract is the best proxy for contagiousness. Therefore, we assessed the feasibility of sampling exhaled air from hospitalized COVID-19 patients to measure SARS-CoV-2 RNA using a modular breath sampler. This enabled the collection of a liquid sample, compatible with the conventional molecular diagnostic infrastructure, using a disposable device compatible with application in a highly infectious surrounding. Additionally, we aimed to determine the influence of patient characteristics on RNA detection and load.

## Materials and methods

### Ethical considerations

This cohort study was conducted between October 2020 and February 2021, according to the principles of Helsinki and the Medical Research Involving Human Subjects Act (WMO) at Radboud University Medical Centre (Radboudumc, Nijmegen, the Netherlands). All study protocols were reviewed and approved by the local ethics board, the Committee on Research Involving Human Subjects (CMO) Arnhem-Nijmegen (CMO 2020–6517), which deemed verbal informed consent sufficient. All participants provided verbal informed consent at inclusion.

### Participants

Patients ≥ 18 years of age were included prospectively within 7 days after admission to the Radboudumc. COVID-19 was confirmed by RT-qPCR on a combined nasopharyngeal throat swab.

### Sample collection

Patient characteristics, clinical features, and routine hemocytometric and inflammatory laboratory measurements were collected on standardized patient charts. Routine diagnostic laboratory measurements were registered on the day of sampling (+ /− 1 day).

From each patient, samples were simultaneously collected using three different methodologies. A combined nasopharyngeal throat swab was taken using the hospital’s protocol according to national guidelines^12^. After collection, the sample was placed in 5.0 mL virus transport medium consisting of Hank’s balanced salt solution (Gibco) containing 2% FCS (Sigma-Aldrich), 100 µg/mL gentamicin (Gibco), and 0.5 µg/mL amphotericin-B (Gibco) and stored at -20 °C until further processing.

**Figure 1:**
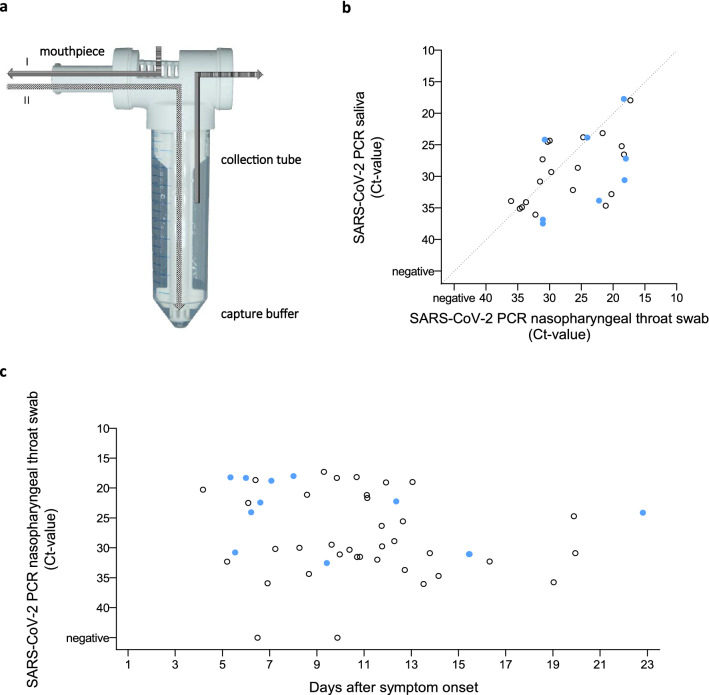
(**a**) Modular breath sampler. During sampling, the patient breathes through the mouthpiece; inhaling (I) and exhaling (II). The exhaled air is guided through the sampler to the diffuser into the capture buffer. The exhaled air leaves the sampler on the back end. After sampling, the collection tube can be disconnected and stored for analysis. (**b**) Correlation plot depicting SARS-CoV-2 viral RNA load measured in nasopharyngeal throat swab and saliva samples. (**c**) SARS-CoV-2 RNA detection in exhaled air samples and nasopharyngeal throat swab samples. Filled dots () represent cases in which SARS-CoV-2 RNA was detected in exhaled air samples. Open dots (○) represent cases in which SARS-CoV-2 RNA was not detected.

Exhaled air was assessed using a modular breath sampler (MBS, Xheal Diagnostics, Fig. 1a). Patients were instructed to inhale and exhale normally through the mouthpiece for one minute, after which they inhaled and exhaled deeply three times before continuing to breath normally to complete two minutes in total. The sample, collected in capture buffer, was stored at − 20 °C until further processing.

Saliva was collected by instructing patients to spit in a sterile 15 or 50 mL container (Greiner) and stored at − 20 °C.

### RT-qPCR

The presence and viral load of SARS-CoV-2 were determined using RT-qPCR adapted from that of the Dutch National Institute for Public Health and the Environment (RIVM). Briefly, 500 µl material was lysed in 450 µl MagNAPure lysis/binding buffer (Roche). RNA internal extraction control (*Plasmodium falciparum* PfMGET ivRNA) was added prior to extraction using the MagNAPure LC Total Nucleic Acid—High Performance kits (Roche). RT-qPCR was performed using the Luna Universal Probe One-Step RTqPCR kit (NEB) with 400 nM E-gene primers (FW: 5’- ACAGGTACGTTAATAGTTAATAGCGT-3’ RV: 5’- ATATTGCAGCAGTACGCACACA-3’) and 200 nM E-gene probe (5’-FAM ACACTAGCCATCCTTACTGCGCTTCG-BHQ1-3’ (Biolegio)) on a CFX96 C1000 Real-Time PCR etection System (BioRad). Transcript quantities were calculated using a tenfold dilution series of E gene ivRNA. The extraction efficiency was checked in a separate RT-qPCR using the Luna Universal Probe One-Step RT-qPCR kit (NEB) with primers targeting PfMGET ivRNA.

### Statistical analyses

Analyses were conducted using SPSS software, 27th version (IBM Corp., 2021) and GraphPad Prism, version 8.0.2 (GraphPad Software, 2019). Categorical data are presented as numbers with percentages and continuous data are presented as medians with interquartile ranges. Mann–Whitney U tests were used to assess differences between groups. Spearman rank correlation was used to assess the correlation between SARS-CoV-2 RNA loads in saliva and nasopharyngeal throat swabs.

## Results

### Patient characteristics

Forty-nine patients (64.4% male) with a median age of 68 years (IQR 52–75) (Table 1) were included. Patients were admitted up to 21 days after symptom onset (median 8, IQR 5–10) and included at a median of 2 days (IQR 2–3.5 days) after admission. Forty-four patients (89.9%) had one or more comorbidities, with cardiovascular diseases including hypertension in 28 (63.3%) and pulmonary diseases including COPD in 16 (36.7%) being the most common. Six patients (12.2%) used immunosuppressive medication prior to SARS-CoV-2 infection.

**Table 1:** Patient characteristics, clinical features and laboratory measurements. Nominal data are presented as numbers with percentages, and continuous data are presented as medians with interquartile ranges. No significant differences were found.

	Total cohort (*n* = 49)	SARS-CoV-2 RNA detected in exhaled air (*n* = 12)	SARS-CoV-2 RNA not detected in exhaled air (*n* = *37)*
**Patient characteristics**			
Sex, % male	34 (69.4%)	9 (75.0%)	25 (67.6%)
Age, years	68 (52–75)	65 (61–73)	69 (51–76)
BMI, kg/m2	28.1 (25.6–31.1)	28.7 (27.4–31.0)	28.1 (25.2–31.2)
Comorbidities	44 (89.8%)	11 (91.7%)	33 (89.2%)
Pulmonary disease	18 (36.7%)	7 (58.3%)	11 (29.7%)
Cardiovascular disease	31 (63.3%)	9 (75.0%)	22 (59.5%)
Diabetes mellitus	11 (22.4%)	3 (25.0%)	8 (21.6%)
Chronic kidney disease	5 (10.2%)	0 (0.0%)	5 (13.5%)
Auto-immune disease	6 (12.2%)	1 (8.3%)	5 (13.5%)
Haematological malignancy	2 (4.1%)	1 (8,2%)	1 (2.7%)
Solid organ malignancy	8 (16.3%)	3 (35.0%)	5 (13.5%)
Solid organ transplantation	1 (2.0%)	0 (0.0%)	1 (2.7%)
Liver disease	2 (4.1%)	1 (8.3%)	1 (2.7%)
HIV/AIDS	0 (0.0%)	0 (0.0%)	0 (0.0%)
Other	22 (44.9%)	5 (41.7%)	17 (45.9%)
Immunosuppressive medication	6 (12.2%)	2 (16.7%)	4 (10.8%)
**Clinical features**			
Time from COVID-19 symptom onset to hospital admission, days	8 (5–10)	6 (4–12)	8 (6–10)
Time from COVID-19 symptom onset to sampling, days	10 (7–13)	8 (6–14)	11 (9–13)
Length of admission, days	7 (4–12.5)	8 (4–14)	7 (4.5–12)
Immunomodulatory COVID-19 treatment			
Corticosteroids	43 (87.8%)	11 (91.7%)	32 (86.5%)
Reason of discharge			
Clinical improvement	36 (73.5%)	8 (66.7%)	28 (75.7%)
Transfer to rehabilitation centre	8 (16.3%)	1 (8.3%)	7 (18.9%)
Patient deceased	5 (10.2%)	3 (25.0%)	2 (5.4%)
**Laboratory measurements**			
Hemoglobin, mmol/L	8.3 (7.5–9.0)	8.2 (7.5–8.9)	8.4 (7.5–9.2)
Thrombocyte count, × 10^9^/L	226 (176–309)	229 (162–277)	226 (185–340)
Leucocyte count, × 10^9^/L	8.2 (6.2–11.1)	7.6 (6.3–10.0)	8.8 (6.2–11.3)
Neutrophil count, × 10^9^/L	6.5 (4.5–9.4)	6.0 (5.3–8.5)	7.6 (4.5–9.6)
Lymphocyte count, × 10^9^/L	0.8 (0.6–1.3)	0.8 (0.6–1.3)	0.8 (0.6–1.4)
Monocyte count, × 10^9^/L	0.5 (0.3–0.7)	0.6 (0.3–0.8)	0.5 (0.3–0.7)
Eosinophil count, × 10^9^/L	0.0 (0.0–0.0)	0.0 (0.0–0.0)	0.0 (0.0–0.0)
Basophil count, × 10^9^/L	0.0 (0.0–0.0)	0.0 (0.0–0.0)	0.0 (0.0–0.0)
C-reactive protein, mg/L	51 (28–99)	71 (35–175)	47 (25–92)
Ferritin, µg/L	959 (583–1594)	939 (648–3061)	959 (550–1541)
D-dimer, µg/L	1505 (795–3502)	2005 (948–3353)	1080 (515–1815)

The majority of patients (87.8%, 43/49) received immunomodulatory treatment for COVID-19 in the form of corticosteroid treatment. Thirty-six patients (73.5%) showed clinical improvement and were discharged, 8 patients (16.3%) were transferred to a rehabilitation center, and 5 patients (10.2%) died within the hospital. Median length of admission was 7 days (ICR 4–12.5).

### Correlation of viral RNA load in saliva and nasopharyngeal throat swabs

Out of 30 patients from whom we collected saliva samples, 27 (90%) had detectable SARS-CoV-2 RNA in both saliva and the combined nasopharyngeal-throat swab. SARS-CoV-2 RNA loads in saliva and the combined nasopharyngeal throat swab were correlated (*r*_*s*_ = 0.566, *p* = 0.002) (Fig. 1b).

### Detection of SARS-CoV-2 RNA in exhaled air

SARS-CoV-2 RNA was detected in exhaled air in 12 (24.5%) patients (Fig. 1c) up to 23 days after symptom onset. Patients with and without detectable SARS-CoV-2 RNA in exhaled air did not differ in age, BMI, time from symptom onset to admission, length of admission, or laboratory measures (Table 1). The median time from symptom onset to sampling was 7.5 days (IQR 6–14.3) in patients with detectable SARS-CoV-2 RNA versus 11 days (IQR 8.5–13) in patients without detectable SARS-CoV-2 RNA (p = 0.295). Interestingly, the presence of SARS-CoV-2 RNA in exhaled air was not limited to patients with high SARS-CoV-2 viral load in the combined nasopharyngeal throat swab. Furthermore, patients with and without detectable SARS-CoV-2 RNA in exhaled air showed no differences in SARS-CoV-2 RNA load in the combined nasopharyngeal throat swab (*p* = 0.335) or saliva (*p* = 0.938, Supplementary Fig. 1).

## Discussion

In this study, we showed for the first time that SARS-CoV-2 RNA can be detected by RT-qPCR in exhaled air of hospitalized COVID-19 patients. SARS-CoV-2 RNA has previously been detected in air collected from COVID-19 wards^13^ and in exhaled air from ambulant COVID-19 patients^14^, but not yet from hospitalized patients. In our study, sampling of exhaled air was feasible using a handheld modular breath sampler and viral RNA was detected in almost 25% of the patients.

We demonstrated that viral RNA is still detectable 7 days after disease onset in a clinical setting, later than Coleman et al.^14^ who detected SARS-CoV-2 RNA after a median of 3 days of symptoms in an ambulant setting. Their positivity rate was higher, which can probably also be attributed to a longer sampling time of 30 min. It must be noted that the two-minute breathing exercise was well tolerated by all patients in our study, including patients receiving oxygen supplementation via nasal cannula as well as patients using high-flow oxygen therapy. The sampling could be prolonged to increase sensitivity, however, increased sampling time will reduce the usability of the modular breath sampler and hamper its clinical implication.

Viral load in nasopharyngeal throat swab and saliva samples was positively correlated, confirming previous observations both in inpatients with confirmed COVID-19^15^ and in routine sampling^16^. Sampling saliva can be a patient-friendly alternative to nasopharyngeal throat swab sampling, but sensitivity can be an issue, as shown by the lower viral loads in saliva.

The fact that SARS-CoV-2 RNA was measured up to 23 days after symptom onset suggests long-term persistence of viral RNA. Moreover, the absence of any association between SARS-CoV-2 positivity in exhaled air samples and viral load in nasopharyngeal throat swab and saliva samples makes contamination from the upper airway unlikely.

This study shows the feasibility of sampling exhaled air from hospitalized COVID-19 patients for the presence of SARS-CoV-2 RNA. The modular breath sampler could be used in epidemiological studies to determine the best proxy of SARS-CoV-2 contagiousness.

## Supplementary Information

Supplementary Information.

## Data Availability

The data that support the findings of this study are available from the corresponding author, upon reasonable request.
