# Supplementary material for: SARS-CoV-2 RNA in exhaled air of hospitalized COVID-19 patients
Source: Sci Rep. 2022 May 30;12:8991. doi: 10.1038/s41598-022-13008-4 (PMC9151771; doi:10.1038/s41598-022-13008-4)
Supplement: Supplementary file 1 — Supplementary Information. [file 41598_2022_13008_MOESM1_ESM.pdf]

Supplementary materials for

SARS-CoV-2 RNA in exhaled air of hospitalized COVID-19 patients

Authors: Lisa Kurver[a], Corné H. van den Kieboom[b,g], Kjerstin Lanke[c], Dimitri A. Diavatopoulos[b], Gijs J. Overheul[c], Mihai G. Netea[a,d], Jaap ten Oever[a], Reinout van Crevel[a], Karin Mulders-Manders[a], Frank L. van de Veerdonk[a], Heiman Wertheim[c], Jeroen Schouten[e], Janette Rahamat-Langendoen[f], Ronald P. van Rij[c], Teun Bousema[c], Arjan van Laarhoven[a*], Marien I. de Jonge[b*]

Affiliations:

[a] Department of Internal Medicine and Radboud Centre for Infectious Diseases, Radboud University Medical Center, 6525 GA, Nijmegen, The Netherlands

[b] Laboratory of Medical Immunology, Radboud Institute for Molecular Life Sciences, Radboud University Medical Center, 6525 GA, Nijmegen, The Netherlands

[c] Department of Medical Microbiology, Radboud Institute for Molecular Life Sciences, Radboud University Medical Center, 6525 GA, Nijmegen, The Netherlands

[d] Department of Immunology and Metabolism, Life and Medical Sciences Institute, University of Bonn, 53115 Bonn, Germany

[e] Department of Intensive Care Medicine, Radboud University Medical Center, 6525 GA, Nijmegen, the Netherlands

[f] Department of Medical Microbiology, Radboud Centre for Infectious Diseases, Radboud University Medical Center, 6525 GA, Nijmegen, the Netherlands

[g] Xheal Diagnostics B.V., 6525 GC Nijmegen, The Netherlands

*These authors contributed equally to this work

Corresponding Author: Dr. Marien I. de Jonge
Radboud University Medical Center

Geert Grooteplein Zuid 10, route 469
6525 GA, Nijmegen, The Netherlands
Telephone number: +31 6 57 58 47 00
E-mail address: Marien.deJonge@radboudumc.nl

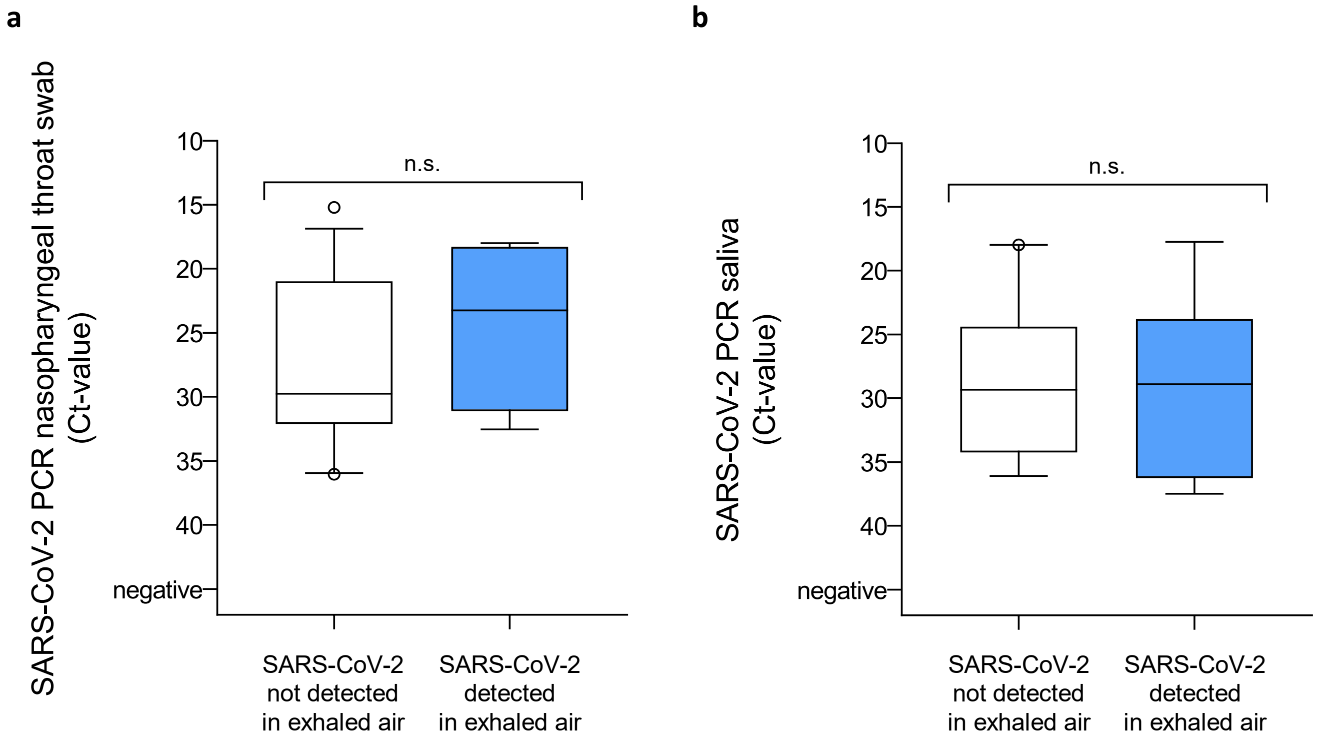

Supplementary Figure 1: a) *Boxplot depicting SARS-CoV-2 viral RNA load measured in nasopharyngeal throat swabs for patients without and with detectable SARS-CoV-2 RNA in the exhaled air sample.* ***b)*** *Boxplot depicting SARS-CoV-2 viral RNA load measured in saliva for patients without and with detectable SARS-CoV-2 RNA in the exhaled air sample. No significant differences were found.*

Filled boxes represent the group in which SARS-CoV-2 RNA was detected in exhaled air samples. Open boxes represent the group in which SARS-CoV-2 RNA was not detected in exhaled air samples.
